# Supplementary material for: Contribution of Autophagy to Cellular Iron Homeostasis and Stress Adaptation in Alternaria alternata
Source: Int J Mol Sci. 2024 Jan 17;25(2):1123. doi: 10.3390/ijms25021123 (PMC10816921; doi:10.3390/ijms25021123)
Supplement: Supplementary file 1 [file ijms-25-01123-s001.zip › ijms-2756044-supplementary.pdf]

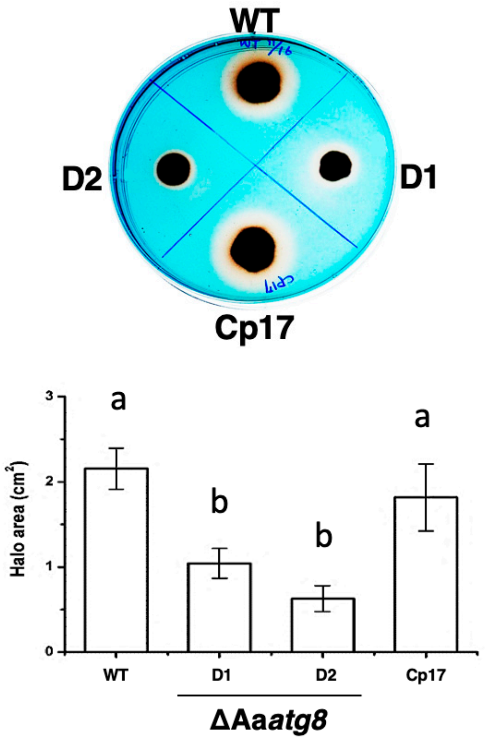

Figure S1. Siderophore production by the *A. alternata* strains. Fungal strains, including the wild type (WT), deletion mutants (ΔAa*atg8*_D1 and D2), and complementation strain (Cp17), were grown on CAS agar medium at 28°C for 5 days. The emergence of an orange halo surrounding the fungal colony indicates siderophore production. The area of orange-yellow halos minus the fungal colony was measured. The data presented are the means of three independent experiments and means with the same letter are not significantly different ($p < 0.05$).

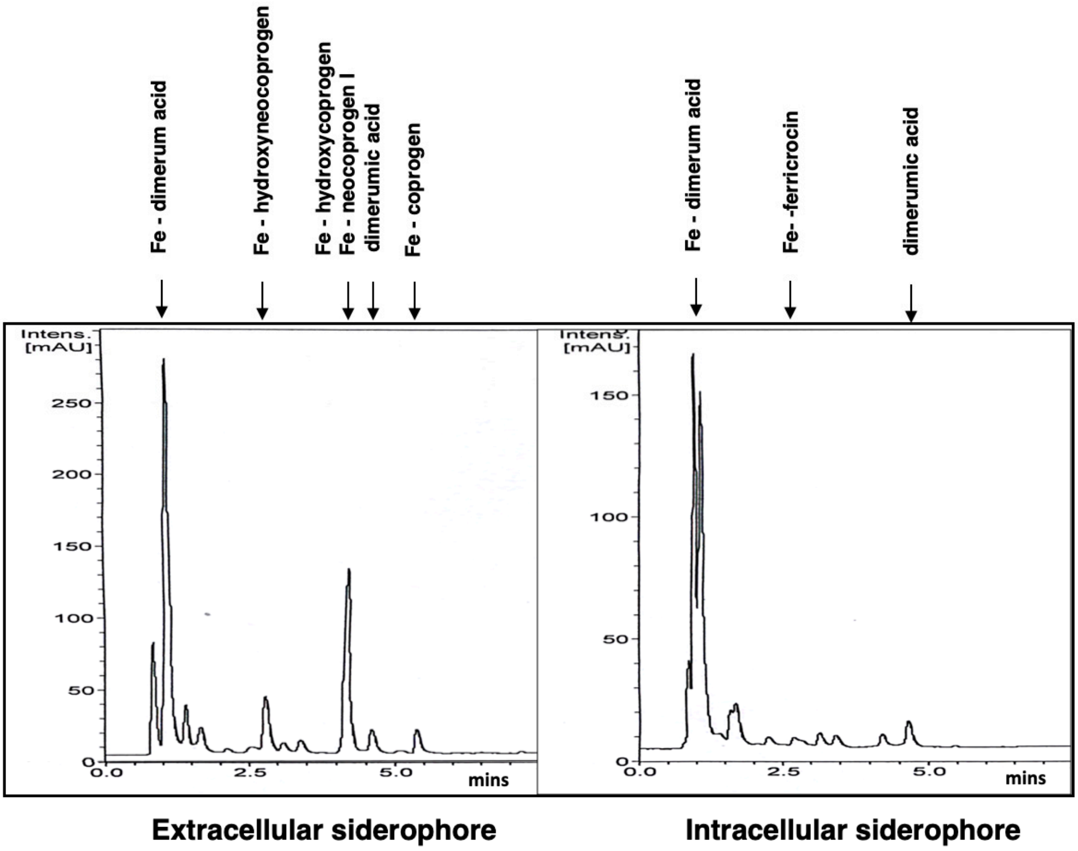

Figure S2. LC-MS/MS analysis was performed to measure intra- and extracellular siderophore produced by wild type. The identities corresponding to different types of siderophores are labeled above the respective peaks.

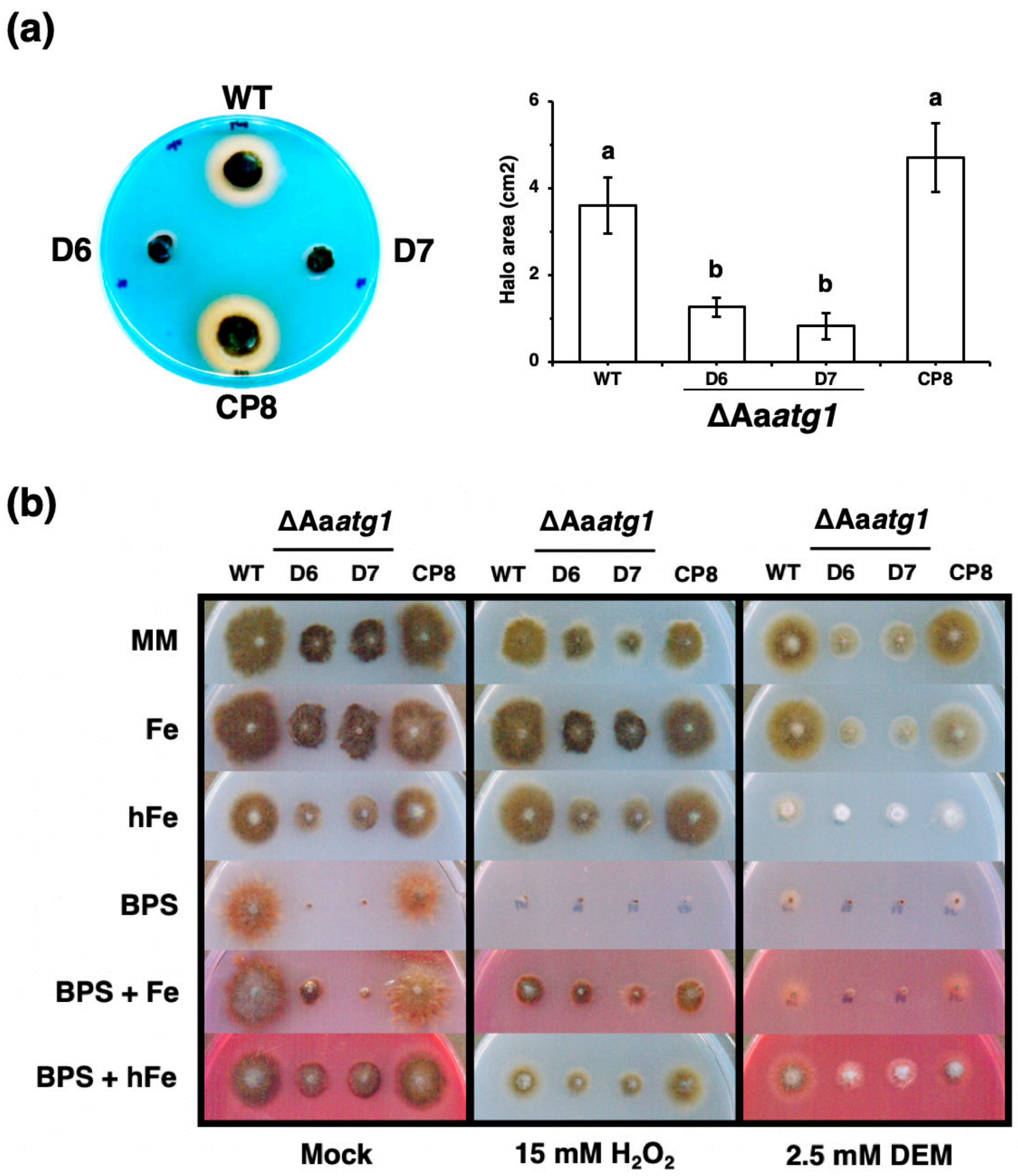

Figure S3. Iron utilization and resistance to oxidative stress in the ΔAa*atg1* strains. (**a**) Fungal strains, including the WT, deletion mutants (ΔAa*atg1*_D6 and D7), and complementation strain (CP8), were grown on CAS agar medium at 28°C for 5 days. (**b**) The WT, ΔAa*atg1* mutants, and CP8 strain were grown on MM or MM supplemented with 25 μM $FeSO_4$ (Fe), 0.5 mM $FeSO_4$ (hFe), 0.25 mM BPS, 15 mM H_2O_2, or 2.5 mM DEM.

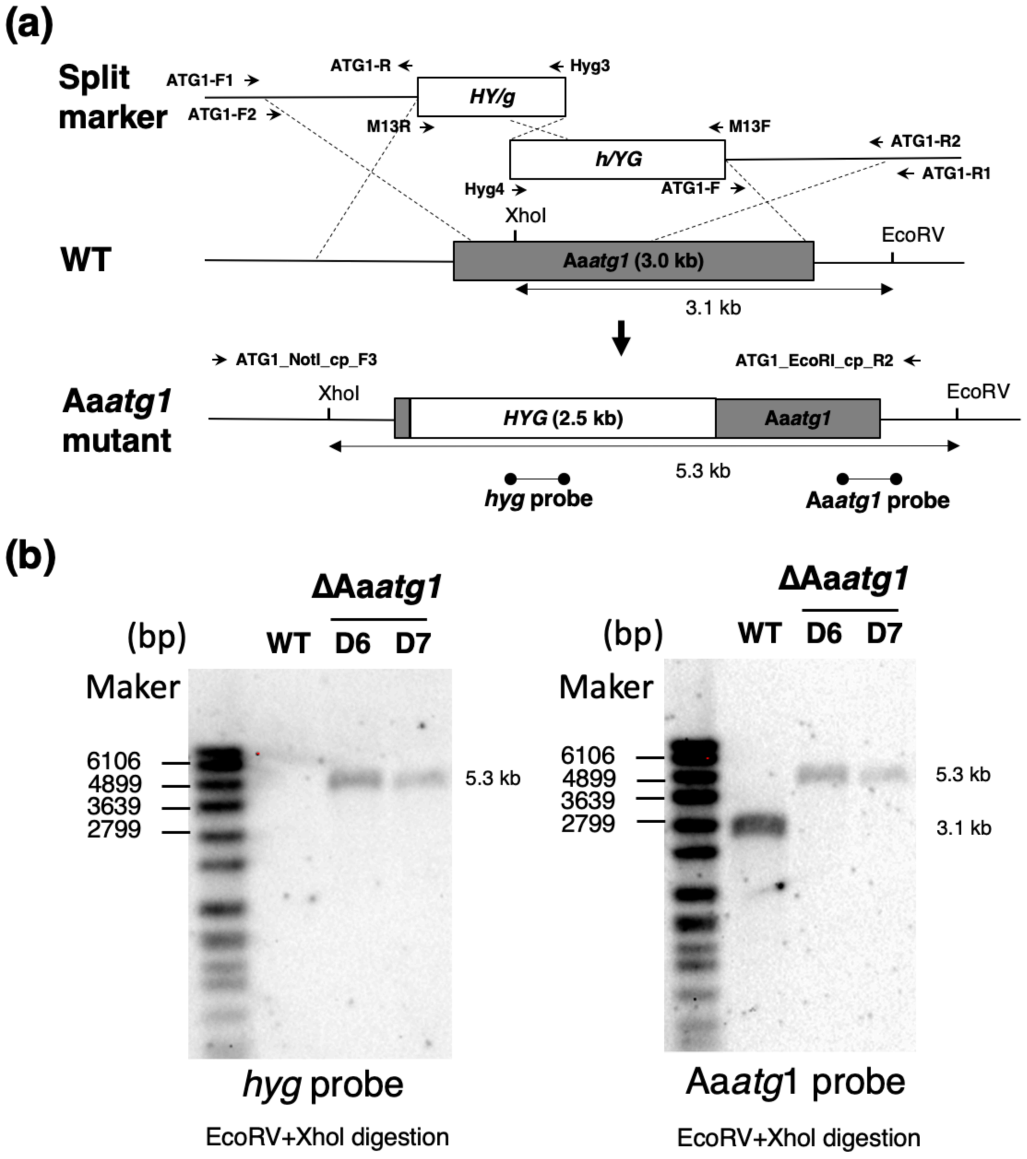

Figure S4. Depict of Aa*atg1* gene disruption in the wild-type EV-MIL31 strain using a split marker strategy. (**a**) Homologous recombination utilized truncated, overlapping DNA fragments from the hygromycin phosphotransferase-coding gene (*hyg*), amplified by PCR with specified primers. (**b**) Southern-blot hybridization of genomic DNA prepared from WT and ΔAa*atg1* mutants (D6 and D7) digested with EcoRV and XhoI, probed with Aa*atg1* or *hyg* probes. The Aa*atg1* probe successfully identified the anticipated 3.1-kb band in the genomic DNA of the WT. In the genomic DNA of the two transformants (D6 and D7), a 5.3-kb band was observed. In addition, using a *hyg*-specific probe, a 5.3-kb band was detected in both D6 and D7 transformants. No band was detected in the wild-type DNA.

Table S1. The oligonucleotide primers were designed for the knockout and variation analysis of the Aa*atg1* gene.

**Primer**	**Sequence (5′→3′)**	**Usage**
ATG1-F1	TTCCGACCATCTTCATCGATCCTTG	Amplification of 5′- and 3′-fragment and Hygromycin resistance gene (*hyg*)-fusion
ATG1-F2	GTGGGATGTCAAGGATAGGGATGTG	
ATG1-R	tcctgtgtgaaattgttatccgCGCTTGAACTTGCCGATAATCTGCTCCGA	
ATG1-F	tcgtgactgggaaaaccctggcATTTGTATTTGTAGAGAAGCGCCGT	
ATG1-R2	AGTACATCAAGCTTTTTCTTCAGCGC	
ATG1-R1	CATTGTCCCCAACCAGACAGAAAGA	
M13F	GCCAGGGTTTTCCCAGTCACGAC	Amplification of *hyg*
M13R	GCGGATAACAATTTCACACAGGA	
Hyg3	GGATGCCTCCGCTCGAAGTA	Amplification of *hyg,* southern blot probe
Hyg4	CGTTGCAAGACCTGCCTGAA	
ATG1_NotI_cp_F3	ttactGCGGCCGCTACGCGCAGAAAGGTGTTCCAT	Amplification of Aa*atg1* gene complementation fragment
ATG1_EcoRI_cp_R2	ggatcGAATTCACATCATCGCTACCCAAGTTCCTG	
ATG1-Southern-F	GCCATTGATGAGCTTACTGGACAC	Aa*atg1* probe for southern blot
ATG1-Southern-R	CATTGTCCCCAACCAGACAGAAAGA	

Table S2. Oligonucleotide primers for qRT-PCR.

**Primer**	**Sequence (5′→3′)**	**Remarks**
Tub2Q-1F	TTCGTCGGTAACTCCACCTCCATC	*β-tubulin*
Tub2Q-1R	ACTCAGCCTCAGTGAACTCCATCTC	
SreA_qPCR_F	CTGGTGGAGGGCGATGTA	*sreA*
SreA_qPCR_R	GAACGCTGGACAGCCACT	
HapX_qPCR_F1	CGATATGGACTGGCGTCAAC	*hapX*
HapX_qPCR_R1	GCTTGCATCTAGCTCCATCG	
Nps6_qPCR_F	TCGCAACTATTATCCGTGGAG	*nps6*
Nps6_qPCR_R	CTGGATGGCCCATATACAATG	
Sit1_qPCR_F1	CGGTATGATTTGCTGCCACA	*sit1*
Sit1_qPCR_R1	ACAGGCATCTACCTCGCTAC	
SidA_qPCR_F	AATGGTATCTTGAGCGAGAACTTT	*sidA*
SidA_qPCR_R	GACACAGATCAGGTCGTGTAATTC	
Ftr1_qPCR_F	CTCCCATTAGAGTGATGATGACG	*ftr1*
Ftr1_qPCR_R	GTCTAGTCATCTGTGCTGGAGTC	
